# Childhood sleep and adolescent chronic fatigue syndrome (CFS/ME): evidence of associations in a UK birth cohort

**DOI:** 10.1016/j.sleep.2018.01.005

**Published:** 2018-06

**Authors:** Simon M. Collin, Tom Norris, Paul Gringras, Peter S. Blair, Kate Tilling, Esther Crawley

**Affiliations:** a Centre for Child and Adolescent Health, University of Bristol, Oakfield House, Oakfield Grove, Bristol, BS8 2BN, UK; b Bristol Medical School, University of Bristol, Canynge Hall, 39 Whatley Road, Bristol, BS8 2PS, UK; c Department of Health Sciences, College of Medicine, Biological Sciences and Psychology, University of Leicester, Centre for Medicine, University Road, Leicester, LE1 7RH, UK; d Evelina London Children's Hospital, Guy's and St Thomas' NHS Foundation Trust, Westminster Bridge Road, London, SE1 7EH, UK

**Keywords:** Paediatric, Adolescence, Chronic fatigue syndrome, CFS/ME, Sleep, ALSPAC

## Abstract

**Objective/Background:**

Sleep abnormalities are characteristic of chronic fatigue syndrome (CFS, also known as ‘ME’), however it is unknown whether sleep might be a causal risk factor for CFS/ME.

**Patients/Methods:**

We analysed data from the Avon Longitudinal Study of Parents and Children (ALSPAC) birth cohort. We describe sleep patterns of children aged 6 months to 11 years, who were subsequently classified as having (or not having) ‘chronic disabling fatigue’ (CDF, a proxy for CFS/ME) between the ages 13 and 18 years, and we investigated the associations of sleep duration at age nine years with CDF at age 13 years, as well as sleep duration at age 11 years with CDF at age 16 years.

**Results:**

Children who had CDF during adolescence had shorter night-time sleep duration from 6 months to 11 years of age, and there was strong evidence that difficulties in going to sleep were more common in children who subsequently developed CDF. The odds of CDF at age 13 years were 39% lower (odds ratio (OR) = 0.61, 95% CI = 0.43, 0.88) for each additional hour of night-time sleep at age nine years, and the odds of CDF at age 16 years were 51% lower (OR = 0.49, 95% CI = 0.34, 0.70) for each additional hour of night-time sleep at age 11 years. Mean night-time sleep duration at age nine years was 13.9 (95% CI = 3.75, 24.0) minutes shorter among children who developed CDF at age 13 years, and sleep duration at age 11 years was 18.7 (95% CI = 9.08, 28.4) minutes shorter among children who developed CDF at age 16 (compared with children who did not develop CDF at 13 and 16 years, respectively).

**Conclusions:**

Children who develop chronic disabling fatigue in adolescence have shorter night-time sleep duration throughout early childhood, suggesting that sleep abnormalities may have a causal role in CFS/ME or that sleep abnormalities and CFS/ME are associated with a common pathophysiological cause.

## Highlights

• Adolescents who develop chronic disabling fatigue (CDF) have shorter nighttime sleep duration throughout early childhood.
• For each additional hour of nighttime sleep at age nine, the odds of CDF at age 13 were 40% lower. For each additional hour of nighttime sleep at age 11, the odds of CDF at age 16 were 50% lower.
• Sleep abnormalities may have a causal role in CFS/ME, or sleep abnormalities and CFS/ME are related to a common pathophysiological cause.

## 1 Background

Chronic fatigue syndrome (CFS, also known as Myalgic encephalomyelitis [ME]) in children and young people is a debilitating disease with an adverse effect on the lives of children and their families [1], [2], [3]. The UK National Institute for Health and Care Excellence (NICE) guidelines state that diagnosis of CFS/ME should be made after three months of persistent or recurrent fatigue that is not the result of ongoing exertion, not substantially alleviated by rest, has resulted in a substantial reduction in activities, has no other known cause and is accompanied by one or more of ten typical symptoms [4]. The widely used CDC/Fukuda diagnostic criteria require six months’ duration of fatigue and the presence of four of eight typical symptoms [5]. Both criteria specify disturbed or unrefreshing sleep as one of the typical symptoms of CFS/ME, and this symptom has been recorded in 95–97% of adult and adolescent (age 12–18 years) patients [6], [7] and in 85% of paediatric patients under 12 years of age [6].

Whilst abnormal sleep is part of the clinical picture of CFS/ME [8], and a major feature of the lived experience of adult [9] and paediatric [10] patients, hypothetical causal pathways that associate sleep dysfunction with CFS/ME have not been substantiated. These include sleep abnormalities and CFS/ME sharing a common cause (physiological, neurological, etc.), CFS/ME precipitating or perpetuating sleep dysfunction or *vice versa*, or abnormal sleep in patients with CFS/ME being a symptom of common CFS/ME-related comorbidities such as low mood and chronic pain [8].

Birth cohort data from the Avon Longitudinal Study of Parents and Children (ALSPAC) have been used to describe childhood sleep patterns from 6 months to 11 years [11] of age and to estimate the prevalence of ‘chronic disabling fatigue’ (CDF, a proxy for clinically diagnosed CFS/ME) at ages 13, 16 and 18 years [12], [13], [14]. In the present study, we describe sleep patterns in children who were subsequently classified as having (or not having) CDF, and we investigate possible associations of sleep at age nine years with CDF at age 13 years and sleep at age 11 years with CDF at age 16 years.

## 2 Methods

### 2.1 Study cohort

ALSPAC is a population-based study that aims to investigate a wide range of influences on the health and development of children [15]. Pregnant women residing in the former Avon Health Authority in South West England, who had an estimated date of delivery between 1 April 1991 and 31 December 1992, were invited to participate, which resulted in a cohort of 14,541 pregnancies and 13,978 children alive at 12 months of age (excluding triplets and quadruplets). The primary source of data for the present study was parent-completed questionnaires administered at four time points during the antenatal period and at regular intervals following birth. The ALSPAC study website contains details of all the data that are available through a fully searchable data dictionary (www.bris.ac.uk/alspac/researchers/data-access/data-dictionary/). Ethical approval for the study was obtained from the ALSPAC Ethics and Law Committee (IRB00003312) and the local research ethics committees.

### 2.2 Classification of CFS/ME

**Fig. 1:**
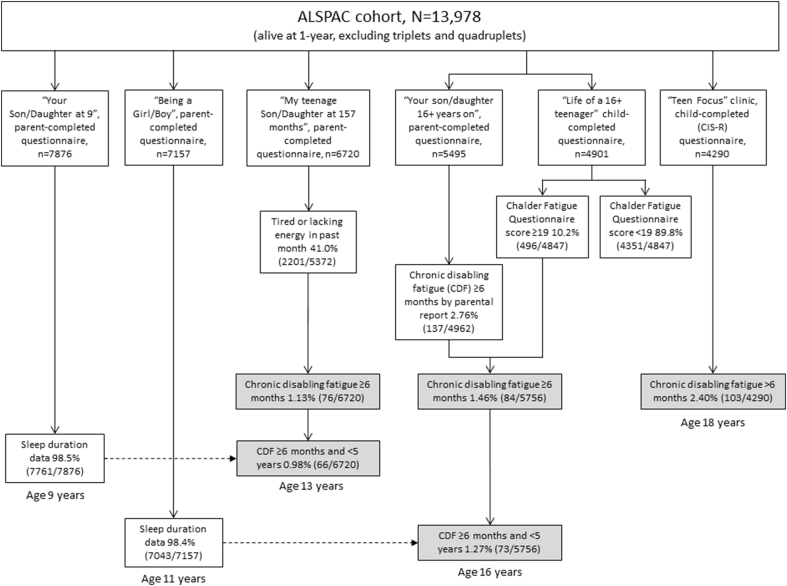
Flowchart of Avon Longitudinal Study of Parents and Children (ALSPAC) participants who provided exposure and outcome data for this study.

We use the term ‘chronic disabling fatigue’ (CDF) rather than CFS/ME because children in our study were not examined by a physician. Children were classified as having CDF (of >6 months’ duration) based on information provided by parents at age 13 years [12], by parents and children at age 16 years [13] and by children at age 18 years [14]. CDF at ages 13 and 16 years was defined as fatigue (feeling tired or lacking in energy) of >6 months’ duration that was associated with absence from full-time school or that had prevented the child from taking part in activities ‘quite a lot’ or ‘a great deal’, excluding fatigue possibly associated with sport, snoring, and other illnesses [12]. At age 16 years, children could only be classified as having CDF if they scored ≥19 (of 33) on the Chalder Fatigue scale [16], [17]. Teenagers completed the Revised Clinical Interview Schedule (CIS-R) [18] at age 18 years and were classified as having CDF if they had been getting tired or had been lacking in energy during the past month and then responded ‘yes’ to >2 of the following four items: (1) felt tired or lacking in energy for four days or more in the past seven days; (2) felt tired or lacking in energy for more than three hours in total on any day in the past seven days; (3) felt so tired or lacking in energy that they had to push themselves to get things done on one or more occasion in the past seven days and (4) felt tired or lacking in energy when doing things they enjoy in the past seven days. Teenagers were not classified as having CDF if the tiredness or lack of energy had lasted for <6 months or was caused by exercise or medication or if the teenager felt better after resting or did not feel exhausted the day after exercising. Children who met CDF criteria at age 16 or 18 years but who were reported by parents to have had problems with alcohol or drugs (crack, solvents, heroin or cocaine) or were diagnosed to have anorexia nervosa were classified as not having CDF. According to our definitions, the prevalence of CDF of >6 months’ duration at age 13, 16 and 18 years was 1.13% (76/6720), 1.46% (84/5756) and 2.40% (103/4290), respectively [14] (Fig. 1). Our definitions of CDF at each age did not exclude children who had comorbid depressive symptoms. Approximately 30% of the children with CFS/ME in specialist services have comorbid depression, and it is unclear whether comorbid depressive symptoms are predictive of, or secondary to, CFS/ME [19]. Therefore, we think it would be unreasonable to reclassify or exclude all children with CDF and comorbid depression, and this would also substantially reduce the study power (halving the prevalence of CDF at some age points). In ALSPAC, high levels of comorbid depressive symptoms in children with CDF at ages 13, 16 and 18 years can be defined using the scores of the Short Mood and Feelings Questionnaire (SMFQ) with a threshold ≥11 [20]. The proportions of children with CDF at age 16 and 18 years who would be excluded from our analysis because of missing SMFQ data were 56% (47/84) and 51.5% (53/103), respectively. Furthermore, of children with CDF who had SMFQ data, 15.8% (12/76) at age 13 years, 60% (21/35) at age 16 years and 40% (20/50) at age 18 years had scores ≥11. Reclassifying these children as not having CDF (or excluding them from our analysis) would result in only 64, 14 and 30 children with CDF at ages 13, 16 and 18 years, respectively.

### 2.3 Sleep measurements

Maternal reports of infant and child sleep at ages 6, 18, 30, 42, 69, 81, 115 and 140 months follow-up time points were collected using postal questionnaires [11]. Most questionnaires were returned within the first few weeks after being sent out, but some allowance was made for slow responses, with windows of 5–12, 18–24, 29–25, 41–47, 68–75, 80–88, 114–122 and 137–147 months for the ages 6, 18, 30, 42, 69, 81, 115 and 140 months’ time points, respectively. Night-time sleep duration was calculated from questions asking what time (to the nearest minute) the infant or child “normally” went to sleep in the evening (bedtime) and woke up in the morning (waking time). Daytime sleep duration (up to age seven years), the number of night-time awakenings (up to age nine years, in response to ‘How often during the night does he/she usually wake?’) and difficulty going to sleep (from age 18 months to 9 years, in response to ‘In the past year has your had difficulty going to sleep?’) were also recorded. Among children who were not subsequently classified as having CDF at any of the three time points (n = 7824), availability of sleep data declined from 93% at age six months to 80% at age 11 years; among children classified as having CDF (n = 242), availability of sleep data declined from 96% at age six months to 86% at age 11 years (Supplementary Table 1).

### 2.4 Statistical analysis

#### 2.4.1 Sleep patterns from the age of 6 months to 11 years in association with CDF during adolescence

We calculated mean (95% CI) night-time, daytime and total sleep durations and mean bedtime and waking time at each follow-up time point, ie at age 6, 18, 30, 42, 69, 81, 115 and 140 months, among two groups of children: those who were classified as having CDF during adolescence (age 13, 16 or 18 years) and those who were not. We tabulated the number of night-time awakenings (as 0, 1 or 2+) and difficulties going to sleep (as responses ‘No, did not happen’, ‘Yes, but did not worry me’) ‘Yes, worried me a bit and Yes, worried me greatly’). We used a nonparametric test for trend over time (from age six months to nine years) in the number of night-time awakenings and difficulty going to sleep. We also coded these two outcomes as binary variables (night-time awakenings = 0 or 1+; difficulty going to sleep = Yes or No) to calculate odds ratios comparing the two groups of children (No CDF, CDF) adjusted for sex and age (at each follow-up time point). We used likelihood ratios to test whether effects of night-time awakening or difficulty going to sleep on the odds of developing CDF changed over time. We estimated the overall mean difference in bedtime and waking times (in minutes) using linear regression adjusted for sex and age (at each follow-up time point).

#### 2.4.2 Association of sleep with CDF adjusted for potential confounders

We conducted two separate analyses to investigate possible causal relationships between sleep duration at age nine years with CDF at age 13 years and between sleep duration at age 11 years with CDF at age 16 years. We did not have a suitable proximal measure of sleep duration to investigate a causal relationship between sleep duration and CDF at age 18 years. We also estimated (using linear regression) the mean difference in night-time sleep duration at 9 and 11 years of age comparing children who did or did not develop CDF at age 13 and 16 years, respectively.

##### 2.4.2.1 Outcomes

The outcomes of the analyses were CDF at age 13 years (median 13.1 (IQR 13.1–13.2) years) and CDF at age 16 years. CDF at age 16 years was based on parental questionnaire data (median age 16.6 (IQR 16.5–16.8) years) and child questionnaire data (median age 16.7 (IQR 16.5–17.1) years). To avoid possible overlap between primary exposures and outcomes in our causal analyses, we excluded children with >5 years’ duration of fatigue at age 13 (10 of 76 children) and 16 (11 of 84 children) years. After these exclusions, 66/6720 children at age 13 years and 73/5756 children at age 16 years were classified as having CDF for causal analyses (Fig. 1).

##### 2.4.2.2 Primary exposures

The primary exposure for CDF at age 13 years was duration of night-time sleep at age nine years (median 9.7 years). The primary exposure for CDF at age 16 years was duration of night-time sleep at age 11 years (median 11.7 years).

##### 2.4.2.3 Other variables

A set of putative risk factors for paediatric CFS/ME were identified based on our earlier work [12], [13], [14], [21] and a review of the literature. To identify possible associations between our exposures and outcome, we identified confounding variables that could potentially bias any observed association. We stipulated that exposures related to CDF at age 13 years must have been collected at or before age nine years. This was to prevent any overlap between outcome and exposure, thereby removing the possibility of reverse causality. For CDF at age 16 years, the exposure cut-off age was 11 years.

The first step in this process was to hold consultative meetings with experts in the fields related to the primary and other exposures (sleep, physical activity, and child and maternal psychopathology) and specialists in paediatric CFS/ME. Consensus from these meetings was encapsulated in the form of directed acyclic graphs (DAGs). DAGs are causal diagrams which provide a method for formalising and clarifying relationships between variables [22], thereby informing the process of building causal models [23]. DAGs are useful for identifying variables that confound the relationship between two variables, thus providing researchers with a set of variables for which adjustment is necessary (or unnecessary) to obtain unbiased estimates of the association between two variables [24]. We refer to the final (logistic regression) model comprising the outcome (CDF as a binary variable), the primary exposure (sleep duration as a continuous variable) and all the identified confounders as the ‘analysis model’.

##### 2.4.2.4 Missing data and multiple imputation

If the analysis model is fitted to a ‘complete case’ dataset, ie dropping participants who have missing data for any of the variables in the model, standard errors will be inflated and bias may arise. If missingness is dependent only on observed data, ie data in the analysis model are ‘missing at random’ (MAR), then multiple imputations can be used to correct this bias. Multiple imputation uses a model based on the analysis model plus auxiliary variables which are selected because of the following: (a) they are associated with the missing variables; (b) they are associated with the missingness of the missing variables and (c) they do not have too much missing data (to ensure stable imputation models which therefore produce reliable estimates).

The number of imputations required to achieve convergence of parameter estimates was determined by checking the estimate of the Monte Carlo error (MCE) in relation to the standard error of the coefficient being estimated. The number of imputations was increased until MCE reached a value which was <10% of the standard error of the estimate. The sample size after imputation was N = 13,978, representing children in the ‘core’ ALSPAC sample who were alive at age one year and who were either a singleton or twin. Multivariable imputation was performed using an imputation sampling method [25], which incorporates all sources of variability and uncertainty in the imputed values. The analysis model is then fitted to the imputed datasets, using Rubin’s rules to combine the estimates into a single estimate that is unbiased (or less biased) by differential losses to follow-up [26].

## 3 Results

### 3.1 Sleep patterns from age 6 months to 11 years

**Fig. 2:**
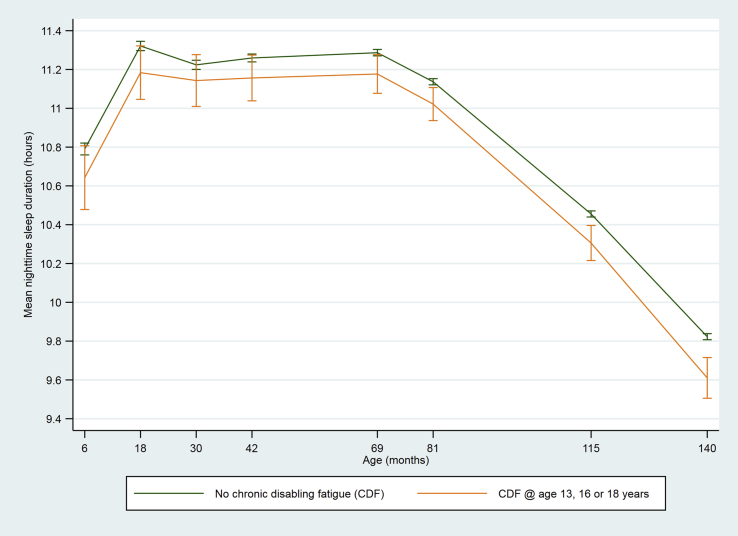
Mean night-time sleep duration at age 6–140 months among children who did or did not develop chronic disabling fatigue (CDF) at age 13, 16 or 18 years (vertical bars indicate 95% CI).

**Fig. 3:**
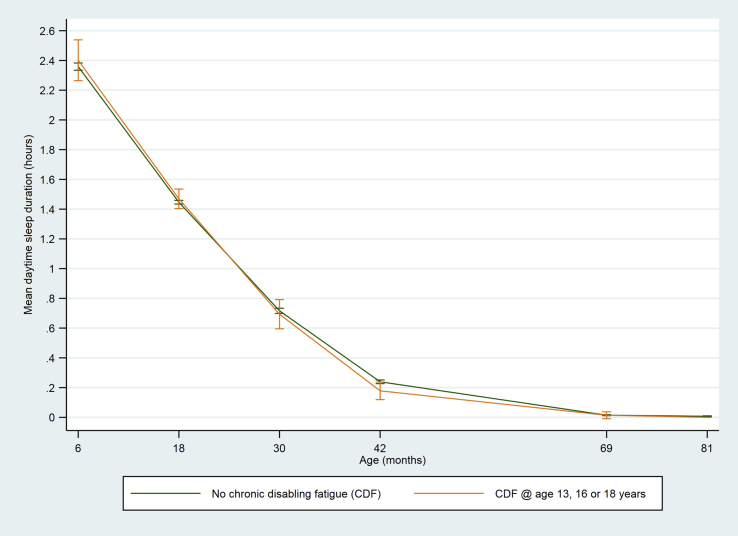
Mean daytime sleep duration at age 6–81 months among children who did or did not develop chronic disabling fatigue (CDF) at age 13, 16 or 18 years (vertical bars indicate 95% CI).

**Fig. 4:**
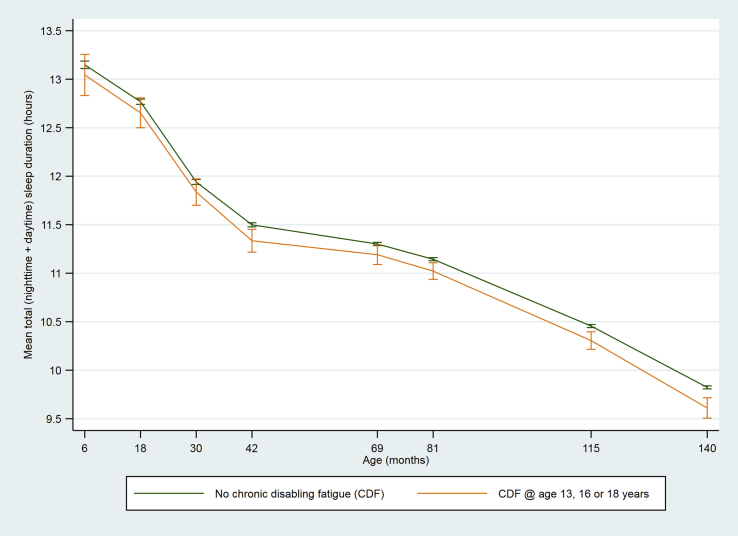
Mean total sleep duration at age 6–140 months among children who did or did not develop chronic disabling fatigue (CDF) at age 13, 16 or 18 years (vertical bars indicate 95% CI).

The total number of children classified with CDF during adolescence was 242, of whom 223 (92.2%) had CDF at only one time point (63 at age 13 years, 67 at age 16 years and 93 at age 18 years) and 19 (7.9%) had CDF at 1 + time points. Children who had CDF during adolescence had shorter night-time sleep duration from age 6 months to 11 years (Table 1, Fig. 2). There were no differences in daytime sleep duration (Table 1, Fig. 3), and the differences in total sleep duration (Table 1, Fig. 4) were attributable to differences in night-time sleep.

**Table 1:** Childhood sleep durations in children with and without chronic disabling fatigue during adolescence (age 13, 16 or 18 years).^a^

	Children without CDF	Children with CDF	Mean difference (95% CI)^b^
	hours (95% CI)	hours (95% CI)	minutes (95% CI)
Night-time sleep duration			
6 months	10.79 (10.76, 10.82)	10.64 (10.48, 10.81)	−8.86 (−19.2, +1.52), p = 0.09
18 months	11.32 (11.30, 11.35)	11.18 (11.05, 11.32)	−8.25 (−16.4, −0.08), p = 0.05
30 months	11.22 (11.20, 11.25)	11.14 (11.01, 11.28)	−4.86 (−12.9, +3.17), p = 0.24
42 months	11.26 (11.24, 11.28)	11.16 (11.04, 11.27)	−6.18 (−13.1, +0.78), p = 0.08
69 months	11.29 (11.27, 11.30)	11.18 (11.08, 11.28)	−6.60 (−12.2, −0.98), p = 0.02
81 months	11.14 (11.12, 11.15)	11.02 (10.94, 11.11)	−6.90 (−12.2, −1.62), p = 0.01
115 months	10.46 (10.44, 10.47)	10.31 (10.22, 10.40)	−8.98 (−14.3, −3.62), p = 0.001
140 months	9.82 (9.81, 9.84)	9.61 (9.51, 9.72)	−12.7 (−18.0, −7.47), p < 0.001
Daytime sleep duration			
6 months	2.358 (2.334, 2.383)	2.401 (2.264, 2.539)	+2.57 (−5.72, +10.9), p = 0.54
18 months	1.447 (1.434, 1.459)	1.469 (1.404, 1.535)	+1.36 (−2.80, +5.52), p = 0.52
30 months	0.716 (0.699, 0.734)	0.693 (0.595, 0.792)	−1.38 (−7.39, +4.63), p = 0.65
42 months	0.239 (0.227, 0.252)	0.178 (0.119, 0.238)	−3.65 (−7.86, +0.56), p = 0.09
69 months	0.014 (0.011, 0.018)	0.014 (−0.009, 0.037)	−0.02 (−1.21, +1.17), p = 0.97
81 months	0.007 (0.005, 0.010)	0.000 (0.000, 0.000)	−0.44 (−1.33, +0.45), p = 0.34
Total sleep duration			
6 months	13.15 (13.11, 13.19)	13.04 (12.83, 13.26)	−6.30 (−19.3, +6.67), p = 0.34
18 months	12.77 (12.74, 12.80)	12.65 (12.50, 12.81)	−6.89 (−16.1, +2.32), p = 0.14
30 months	11.94 (11.91, 11.97)	11.84 (11.70, 11.97)	−6.23 (−14.9, +2.42), p = 0.16
42 months	11.50 (11.48, 11.52)	11.33 (11.22, 11.45)	−9.83 (−17.1, −2.56), p = 0.008
69 months	11.30 (11.28, 11.32)	11.19 (11.09, 11.29)	−6.62 (−12.3, −0.92), p = 0.02
81 months	11.14 (11.13, 11.16)	11.02 (10.94, 11.11)	−7.34 (−12.7, −2.01), p = 0.007
115 months	10.45 (10.44, 10.47)	10.31 (10.22, 10.40)	−8.98 (−14.3, −3.62), p = 0.001
140 months	9.82 (9.81, 9.84)	9.61 (9.51, 9.72)	−12.7 (−18.0, −7.47), p < 0.001

a Based on raw (non-imputed) data – see Supplementary Table 1 for number of observations at each age.

b Student t test.

**Fig. 5:**
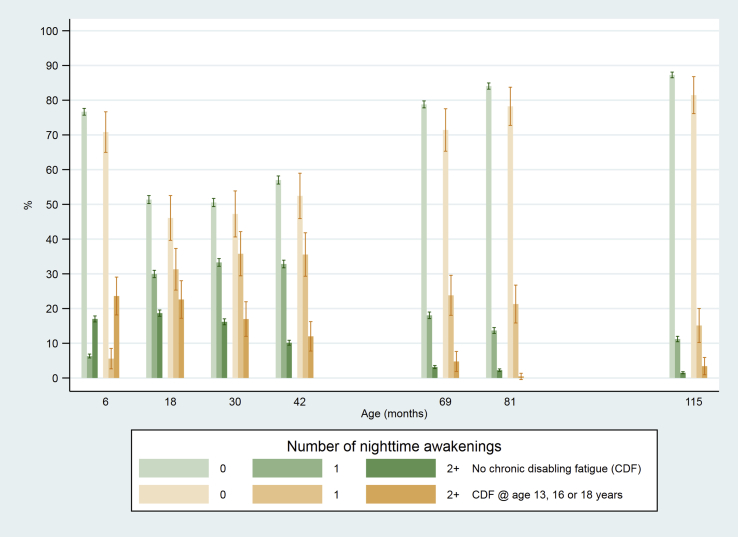
Number of night-time awakenings at age 6–115 months among children who did or did not develop chronic disabling fatigue (CDF) at age 13, 16 or 18 years (vertical bars indicate 95% CI).

In both groups, the number of night-time awakenings decreased as the child grew older (non-parametric test for trend, p < 0.001). There was some evidence that children who developed CDF had more night-time awakenings at ages six months and six, seven and nine years (Fig. 5, Table 2). Overall, the odds of developing CDF were 33% higher among children who had one or more night-time awakenings (odds ratio (OR) = 1.33 (95% CI 1.19–1.49) adjusted for sex and age), and this odds ratio was constant over time (likelihood ratio test for interaction between CDF and time, p = 0.75).

**Table 2:** Childhood night-time awakenings and difficulty in sleeping in children with and without chronic disabling fatigue at age 13, 16 or 18 years.

	Children without CDF				Children with CDF				P-value^a^
	Number of night-time awakenings				Number of night-time awakenings				
	n	0	1	2+	n	0	1	2+	
6 months	7270	5574 (76.7%)	460 (6.33%)	1236 (17.0%)	233	165 (70.8%)	13 (5.58%)	55 (23.6%)	0.03
18 months	7168	3682 (51.4%)	2147 (30.0%)	1339 (18.7%)	230	106 (46.1%)	72 (31.3%)	52 (22.6%)	0.20
30 months	6967	3521 (50.5%)	2319 (33.3%)	1127 (16.2%)	218	103 (47.3%)	78 (35.8%)	37 (17.0%)	0.63
42 months	6951	3963 (57.0%)	2282 (32.8%)	706 (10.2%)	225	118 (52.4%)	80 (35.6%)	27 (12.0%)	0.37
69 months	6421	5059 (78.8%)	1159 (18.1%)	203 (3.16%)	210	150 (71.4%)	50 (23.8%)	10 (4.76%)	0.04
81 months	6440	5414 (84.1%)	881 (13.7%)	145 (2.25%)	216	169 (78.2%)	47 (21.8%)	b	0.002
115 months	6240	5446 (87.3%)	700 (11.2%)	94 (1.51%)	205	167 (81.5%)	31 (15.1%)	7 (3.41%)	0.02
	Child has difficulty going to sleep				Child has difficulty going to sleep				
	n	No	Yes, not worried	Yes, a bit or very worried	n	No	Yes, not worried	Yes, a bit worried	P-value^a^
18 months	7230	5087 (70.4%)	1388 (19.2%)	755 (10.4%)	232	139 (59.9%)	53 (22.8%)	40 (17.2%)	0.001
30 months	6950	4275 (61.5%)	1859 (26.8%)	816 (11.7%)	219	111 (50.7%)	67 (30.6%)	41 (18.7%)	0.001
42 months	7001	4493 (64.2%)	1932 (27.6%)	576 (8.23%)	226	126 (55.8%)	64 (28.3%)	36 (15.9%)	<0.001
69 months	6556	3606 (55.0%)	2356 (35.9%)	593 (9.06%)	215	89 (41.4%)	90 (41.9%)	36 (16.7%)	<0.001
81 months	6560	2428 (37.0%)	3473 (52.9%)	659 (10.1%)	220	43 (19.6%)	128 (58.2%)	49 (22.3%)	<0.001
115 months	6484	3119 (48.1%)	2687 (41.4%)	678 (10.5%)	212	61 (28.8%)	106 (50.0%)	45 (21.2%)	<0.001

a Chi-square test (degrees of freedom = 2).

b ALSPAC does not permit reporting of frequencies <5 – in this row, 47 children had 1+ night-time awakenings (<5 had 2 + awakenings).

**Fig. 6:**
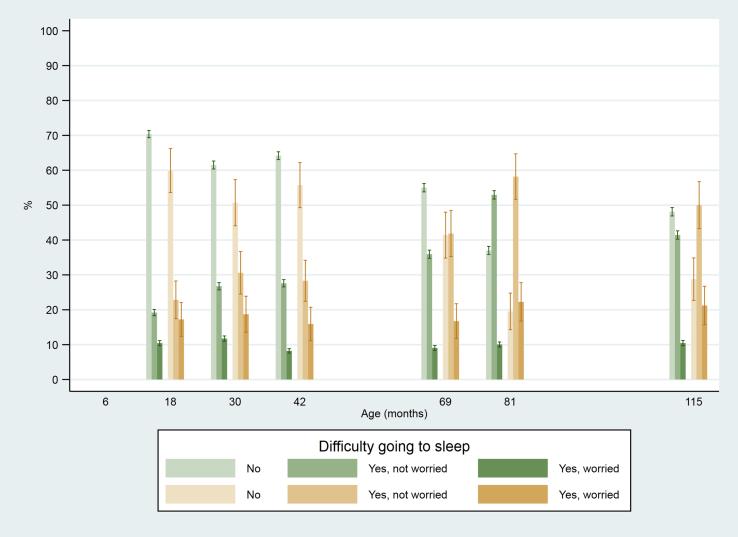
Difficulty going to sleep at age 18–115 months among children who did or did not develop chronic disabling fatigue (CDF) at age 13, 16 or 18 years (vertical bars indicate 95% CI).

Difficulties going to sleep increased in both groups as the child grew older (non-parametric test for trend, p < 0.001). There was strong evidence that difficulties in going to sleep were more common at all ages in children who subsequently developed CDF (Fig. 6, Table 2). The odds of developing CDF were 74% higher among children who had difficulty going to sleep (OR = 1.74 (95% CI 1.55–1.95) adjusted for sex and age). This association increased over time (likelihood ratio test for interaction between difficulty and time, p = 0.07), from 58% higher odds at age 18 months (OR = 1.58 (95% CI 1.21–2.07)) to > twofold higher odds at age nine years (OR = 2.27 (95% CI 1.68–3.06)). Among children who had difficulties going to sleep, those whose mothers were either a bit or very worried were 66% more likely to subsequently develop CDF (OR = 1.66 (95% CI 1.41–1.94)). Moreover, this association did not change over time (likelihood ratio test for interaction between CDF and time, p = 0.67).

**Fig. 7:**
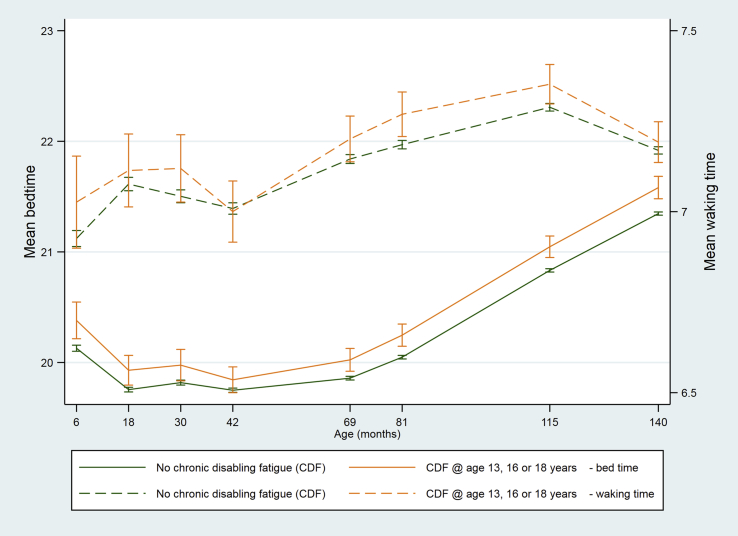
Mean bedtime and waking time at age 6–140 months among children who did or did not develop chronic disabling fatigue (CDF) at age 13, 16 or 18 years (vertical bars indicate 95% CI).

Shorter night-time sleep durations in children who subsequently developed CDF were mainly a consequence of later bedtimes because waking times were similar in the two groups (Table 3, Fig. 7). Overall, children who developed CDF went to bed 11 min later (mean difference = 11.3 (95% CI 8.9 to 13.7) minutes, adjusted for sex and age, p < 0.001) and woke two minutes later (mean difference = 2.4 (95% CI 0.5 to 4.3) minutes, adjusted for sex and age, p = 0.01).

**Table 3:** Childhood bedtimes and waking times (95% CI) in children with and without chronic disabling fatigue at age 13, 16 or 18 years.^a^

	Children without CDF		Children with CDF	
	Bedtime	Waking time	Bedtime	Waking time
6 months	20:08 (20:06, 20:09)	06:56 (06:54, 06:57)	20:22 (20:12, 20:32)	07:01 (06:53, 07:09)
18 months	19:45 (19:44, 19:47)	07:05 (07:03, 07:07)	19:55 (19:47, 20:03)	07:06 (07:00, 07:12)
30 months	19:49 (19:48, 19:50)	07:03 (06:59, 07:05)	19:58 (19:50, 20:07)	07:07 (07:01, 07:12)
42 months	19:45 (19:44, 19:46)	07:01 (07:00, 07:02)	19:50 (19:43, 19:57)	07:00 (06:54, 07:05)
69 months	19:52 (19:51, 19:53)	07:09 (07:08, 07:10)	20:01 (19:55, 20:07)	07:12 (07:08, 07:15)
81 months	20:03 (20:02, 20:04)	07:11 (07:10, 07:12)	20:14 (20:08, 20:20)	07:16 (07:12, 07:19)
115 months	20:50 (20:49, 20:51)	07:17 (07:17, 07:18)	21:02 (20:56, 21:08)	07:21 (07:17, 07:24)
140 months	21:21 (21:20, 21:22)	07:10 (07:10, 07:11)	21:34 (21:28, 21:40)	07:11 (07:08, 07:14)

a Based on raw (non-imputed) data – see Supplementary Table 1 for number of observations at each age.

### 3.2 Associations of sleep duration with CDF

#### 3.2.1 Sleep at age nine years and CDF at age 13 years

For each additional hour of night-time sleep at age nine years, the odds of CDF at age 13 years were 39% lower (odds ratio (OR) = 0.61 (95% CI 0.43, 0.88)) (Table 4). Mean night-time sleep duration at age nine years was 13.9 (95% CI 3.75, 24.0) minutes shorter among children who developed CDF at age 13 years compared to children who did not develop CDF at this age. Effects were similar in girls and boys, ie there was no interaction between sex and sleep duration, and were adjusted for multiple confounders (see footnote to Table 4).

**Table 4:** Associations of night-time sleep duration with chronic disabling fatigue (CDF) at ages 13 and 16 years.

		Raw data	Imputed data^a^^d^
		Odds ratio (95% CI) per hour of sleep	Odds ratio (95% CI) per hour of sleep
CDF at 13 years, night-time sleep at 9 years	Unadjusted	0.53 (0.36, 0.79)	0.53 (0.37, 0.76)
	Partially adjusted^b^	0.52 (0.35, 0.78)	0.52 (0.36, 0.76)
	Fully adjusted^c^	–	0.61 (0.43, 0.88)
CDF at 16 years, night-time sleep at 11 years	Unadjusted	0.45 (0.31, 0.65)	0.44 (0.31, 0.63)
	Partially adjusted^#^	0.45 (0.30, 0.66)	0.44 (0.31, 0.63)
	Fully adjusted^c^^d^	–	0.49 (0.34, 0.70)

a For CDF @ 13 y, auxiliary variables for the multiple imputation were: CDF @ 13 y & 16 y; night-time sleep duration @ 7 y & 9 y; sex; BMI @ 7 y & 9 y; child mood @ 9 y (Short Moods and Feelings Questionnaire score); maternal depression @ 6 y (Edinburgh Postnatal Depression Scale); maternal anxiety @ 6 y (Crown-Crisp Experiential Index); mean test score for English, Mathematics and Science @ 11 y (Key Stage 2 tests); maternal life events score (antenatal); self-esteem @ 8 y (Global Self Worth subscale from Harter’s Self Perception Profile for Children); ALSPAC family adversity index (antenatal); family adversity index @8–10 y; life difficulties @ 11 y (Strengths and Difficulties Questionnaire); screen time @ 6 y; conduct problems @ 8 y; duration of breastfeeding; family income @ 4 y; maternal childhood socio-economic status; vigorous physical activity @ 8 y; maternal education; maternal age at birth of child; maternal psychopathology @ 8-10 y; experienced bullying @ 8 y; days spent outdoors @ 8 y; poor concentration at school @ 7 y; maternal and paternal BMI @ 8 y; internalising behaviour @ 4 y (Strengths and Difficulties Questionnaire). In a sensitivity analysis based on imputation with a different set of (34) variables, the crude and partially adjusted odds ratios were 0.53 (0.38, 0.74) and 0.53 (0.38, 0.74), respectively.

b Adjusted for sex and family adversity index (antenatal).

c Adjusted for: sex; family adversity index (antenatal); child mood @ 9 y; conduct problems @ 8 y; duration of breastfeeding; family income @ 4 y; maternal childhood socio-economic status; maternal life events score (antenatal); maternal depression @ 6 y; maternal anxiety @ 6 y; BMI @ 7 y & 9 y; vigorous physical activity @ 8 y; screen time @ 6 y; experienced bullying @ 8 y; self-esteem @ 8 y; internalising behaviour @ 4 y; maternal and paternal BMI @ 8 y (see Supplementary Fig. 1).

d For CDF @ 16 y, child mood @ 10 y was substituted for mood @ 9 y, night-time sleep duration @ 9 y & 11 y was substituted for sleep duration @ 7 y & 9 y, conduct problems @ 9 y was substituted for conduct @ 8 y and experienced bullying @ 9 y was substituted for experienced bullying @ 8 y (see Supplementary Fig. 2). CDF @ 18 y was also added as an auxiliary variable for the multiple imputations.

#### 3.2.2 Sleep at age 11 years and CDF at age 16 years

For each additional hour of night-time sleep at age 11 years, the odds of CDF at age 16 years were 51% lower (OR = 0.49 (95% CI 0.34, 0.70)) (Table 4). Mean night-time sleep duration at age 11 years was 18.7 (95% CI 9.08, 28.4) minutes shorter among children who developed CDF at age 16 years compared to children who did not develop CDF at this age. Effects were similar in girls and boys, ie there was no interaction between sex and sleep duration, and were adjusted for multiple confounders (see footnote to Table 4).

## 4 Discussion

We have shown that children who developed CDF at age 13, 16 or 18 years had shorter night-time sleep duration from age 6 months to 11 years, mainly because of later bedtimes. Differences in the frequency of night-time awakenings were evident only at age six months and at six, seven and nine years, but there was strong evidence at all ages that difficulties in going to sleep were more common in children who subsequently developed CDF. For each additional hour of night-time sleep at age nine years, the odds of CDF at age 13 years were 39% lower (95% CI 12%–57% lower), and for each additional hour at age 11 years, the odds of CDF at age 16 years were 51% lower (30%–66% lower). The mean differences in sleep duration at age 9 and 11 years, comparing children who did or did not develop CDF at 13 and 16 years, were 14 and 19 min, respectively. These findings suggest that sleep may play a causal role in CFS/ME or that sleep abnormalities share a common cause with CFS/ME.

### 4.1 Strengths and weaknesses

Our study is the first to investigate a temporal relationship between sleep and chronic disabling fatigue (as a proxy for CFS/ME) in adolescence. The main strengths of our study are that it used prospectively collected data from a large population-based birth cohort and that data for exposures, outcomes and potential confounders were collected at multiple time points. Using these data in conjunction with directed acyclic graphs enabled us to examine temporal relationships and to adjust for known measured confounders [22]. Furthermore, we used multiple imputations to adjust for higher losses to follow-up, which occur among families experiencing greater social adversity [15]. The identical odds ratios estimated from the raw (missing data) and imputed datasets are indicative of little or no bias caused by differential losses to follow-up.

The main limitation of our study is that children were not assessed by a doctor, which is why we describe our outcome as ‘chronic disabling fatigue’, a proxy for chronic fatigue syndrome/ME. At age 13 years, our classification was based on parental report of fatigue [12], whereas at age 16 years, we combined parental data with child-completed Chalder Fatigue Questionnaire (CFQ) data [13]. We classified children as not having CDF if they had a CFQ score <19, a threshold which has 82.4% sensitivity and 86.4% specificity for CFS/ME in adults [17]. Our definition of CDF did not exclude children with comorbid depressive symptoms because one-third of the children with CFS/ME in specialist services have comorbid depression, and it is not yet known whether this is a predictor of, or secondary to, CFS/ME [19].

Most of the children classified with CDF at age 13 and 16 years had been fatigued for <5 years (87%), which gives some reassurance that the observed associations between sleep and CDF did not reflect children with a long history of tiredness caused by sleep problems. We think it unlikely that the observed differences, eg around 10 min less night-time sleep at age 9 and 11 years, indicate a primary sleep disorder, but we did not have measurements of sleep at older ages to confirm this. Even if these relatively slight differences in sleep continued into adolescence, they would not be sufficient to explain chronic fatigue resulting in absence from school or preventing the child from taking part in activities, or which was scored ≥19 on the CFQ. We also excluded from our definition fatigue possibly associated with snoring.

The sleep durations in our study were obtained from parents’ (mostly mothers’) answers to questions about the child’s usual bedtime and waking time, rather than from data collected in a sleep/wake diary or by actigraphy. We showed previously in the same cohort that maternal anxiety had a positive association with increased risk of CDF at age 13 years [21], and mothers who experience anxiety might be more likely to report sleep problems in their child. However, we adjusted for maternal anxiety in our analysis, along with a large number (17) of other confounders. This earlier study had shown that childhood psychological problems and upsetting life events (before age nine years) were not associated with CDF at age 13 years after adjusting for maternal anxiety, and we did not include childhood trauma as a potential confounder (although confounders identified by DAG methodology included being bullied, conduct problems and measures of self-esteem, and internalising behaviour).

### 4.2 Our findings in the context of the studies

To our knowledge, all other studies to date of the relationship between sleep and CFS/ME have focused on differences between current patients and healthy controls. Snodgrass et al. identified five case–control and one case series study in a systematic review of research into sleep disturbances in paediatric CFS/ME [10]. All the studies were small (N = 3–57), and the only consistent findings were of increased sleep disturbances in children with CFS/ME, rather than differences in total sleep duration or sleep latency. Jackson and Bruck reviewed the larger body of literature around sleep abnormalities in adult CFS/ME patients [8]. There were few consistent findings, other than discrepancies between subjectively reported and objectively measured sleep, some evidence of differences in sleep stage transitions and instability, and possible implication of heart rate variability and altered cortisol profiles as underlying mechanisms.

One possible explanation for our findings is that there is a heritable difference in a physiological mechanism, which increases susceptibility to CFS/ME [27] and which also affects sleep patterns in such a way that children tend to have later bedtimes. Putative common causes for abnormal sleep and increased CFS/ME susceptibility revolve around the hypothalamic–pituitary–adrenal (HPA) axis function [28], [29], manifesting as differences in cortisol [30], [31], adrenocorticotropic hormone [32] and melatonin [33], and around the autonomic nervous system function, manifesting as differences in heart rate variability [34], [35], [36], [37].

A more direct causal hypothesis is that the children who developed CDF had shorter sleep because they had an ‘evening preference’ chronotype. Thus, their bedtimes were significantly later, and their morning waking times would have been significantly later if not constrained by school start times. Circadian rhythmicity and sleep homeostasis are becoming increasingly well understood and have genetic determinants. Different polymorphisms in the clock gene PER3 are associated with individual morning or evening preferences [38]. These polymorphisms also confer different abilities to cope with sleep deprivation in relation to cognitive performance [39] and vulnerability to adverse psychological effects including mood and state anxiety in the evening preference group [40]. On a population level, there have been numerous reports of genetic associations between these polymorphisms in clock genes and psychiatric disorders (including major depressive disorder, bipolar and seasonal affective disorder) [41], [42].

## 5 Conclusions and future work

We conclude that increased susceptibility to CFS/ME during adolescence may be associated with the function of a physiological mechanism that adversely affects sleep from a very early age. Future work should include detailed comparison of genetic variants in core clock genes in CFS/ME cases compared to controls. This type of investigation would require a large sample of clinically diagnosed cases but has the advantage of being able to use existing data from population controls and to explore differences across the genome.

## Funding

This work was supported by the UK Medical Research Council [grant number MR/K020269/1]. Esther Crawley is funded by an NIHR Senior Research Fellowship (SRF-2013-06-013).
