# Supplementary figures and images for: Childhood sleep and adolescent chronic fatigue syndrome (CFS/ME): evidence of associations in a UK birth cohort
Source: Sleep Med. 2018 Jun;46:26–36. doi: 10.1016/j.sleep.2018.01.005 (PMC5974860; doi:10.1016/j.sleep.2018.01.005)

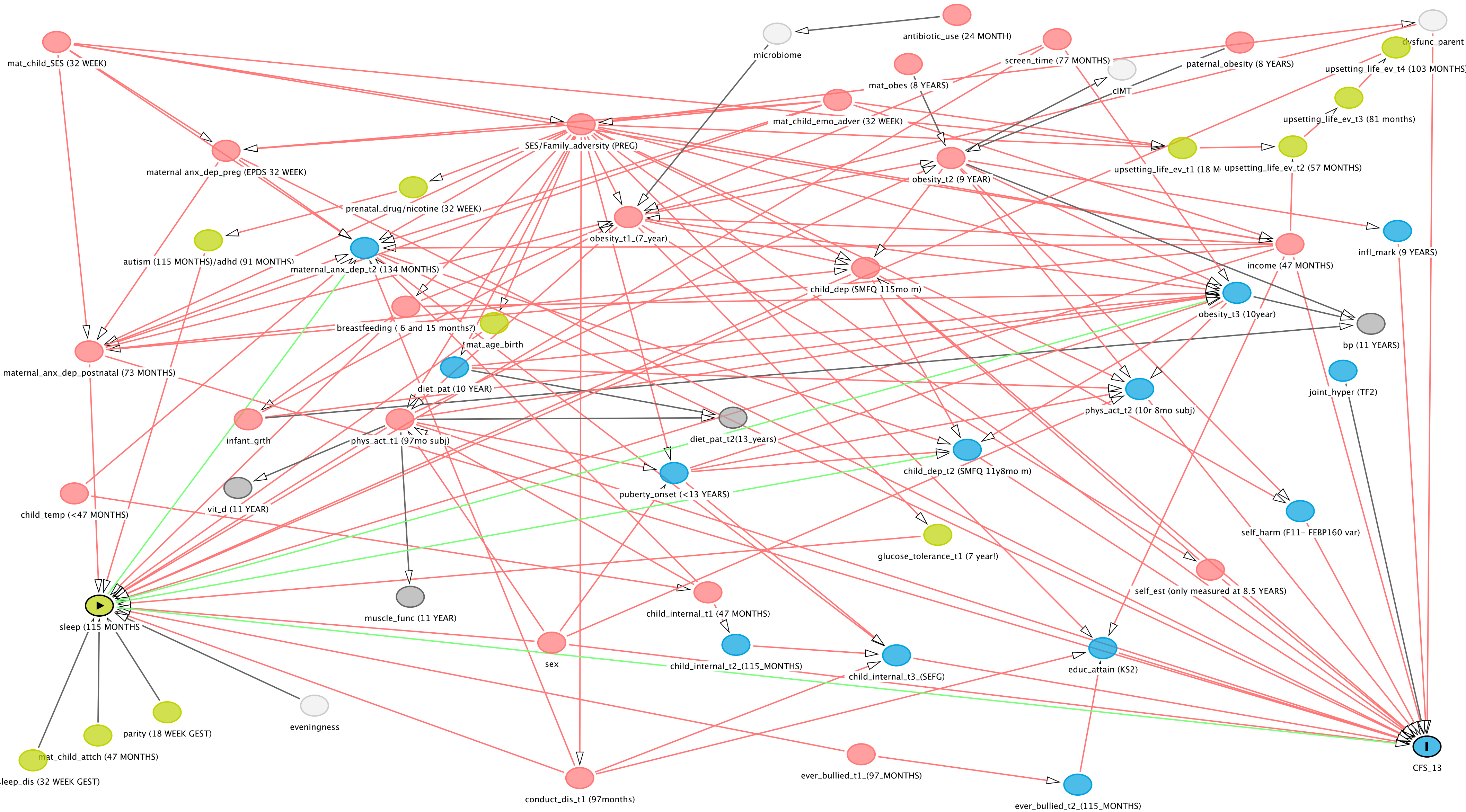

Supplement: Supplementary file 1 — Supplementary Fig. 1 [file mmc1.pdf]

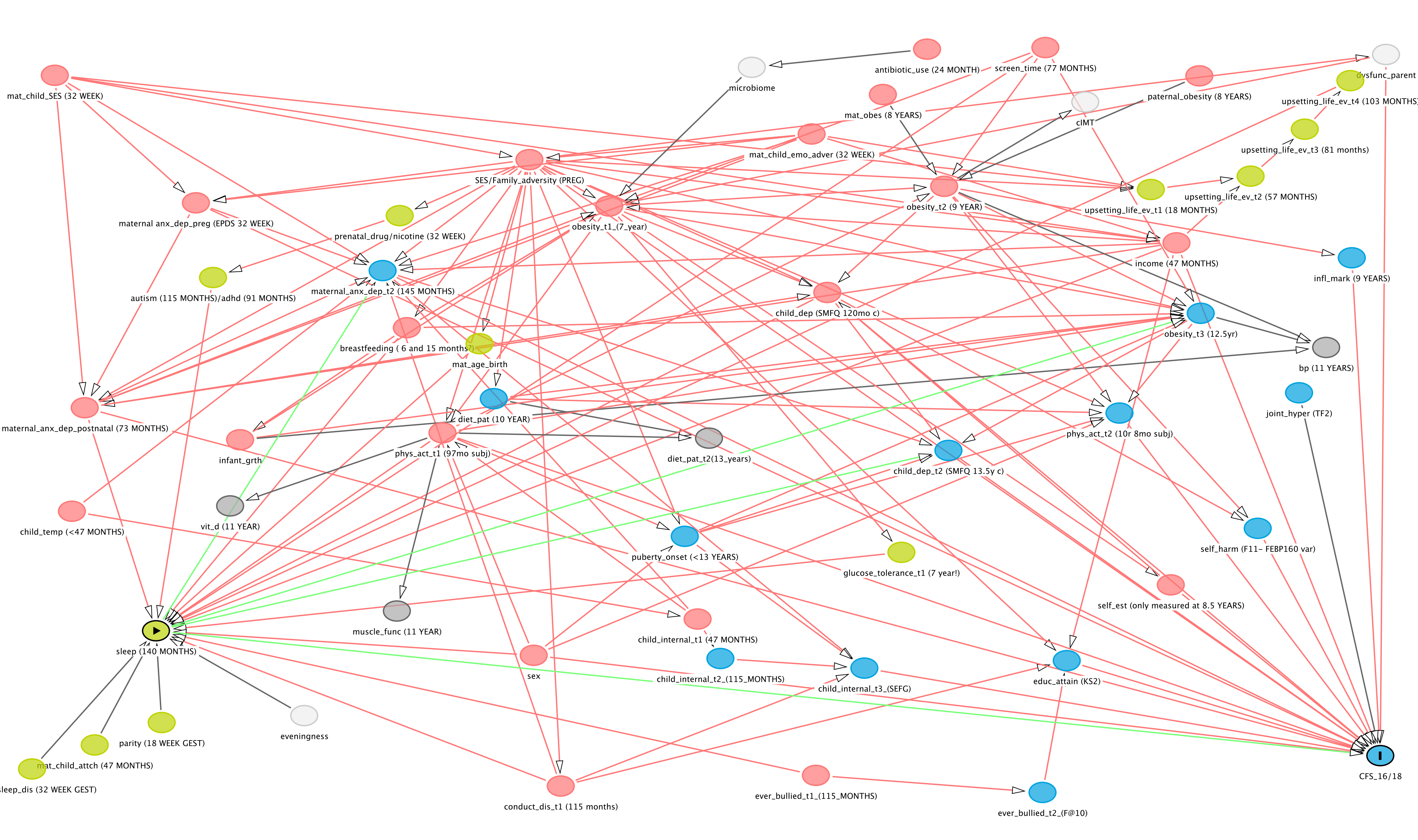

Supplement: Supplementary file 2 — Supplementary Fig. 2 [file mmc2.pdf]
