# Supplementary material for: Childhood sleep and adolescent chronic fatigue syndrome (CFS/ME): evidence of associations in a UK birth cohort
Source: Sleep Med. 2018 Jun;46:26–36. doi: 10.1016/j.sleep.2018.01.005 (PMC5974860; doi:10.1016/j.sleep.2018.01.005)
Supplement: Supplementary Table 1 [file mmc3.docx]

Supplementary Table 1: Availability of sleep data (nighttime duration) at each follow up time point in children who were classified with and without chronic disabling fatigue (CDF) at age 13, 16 and/or 18 years

|  | Follow up time point (months since birth) | | | | | | | |
| --- | --- | --- | --- | --- | --- | --- | --- | --- |
|  | 6 | 18 | 30 | 42 | 69 | 81 | 115 | 140 |
| No CDF at any age (n=7824) | 7254 (92.7%) | 7096 (90.7%) | 6596 (84.3%) | 6738 (86.1%) | 6523 (83.4%) | 6425 (82.1%) | 6502 (83.1%) | 6247 (79.8%) |
| CDF at 13 (n=76) | 73 (96.1%) | 72 (94.7%) | 70 (92.1%) | 70 (92.1%) | 73 (96.1%) | 70 (92.1%) | 71 (93.4%) | 73 (96.1%) |
| CDF at 16 (n=84) | 80 (95.2%) | 78 (92.9%) | 73 (86.9%) | 73 (86.9%) | 72 (85.7%) | 77 (91.7%) | 70 (83.3%) | 71 (84.5%) |
| CDF at 18 (n=103) | 90 (97.1%) | 90 (97.1%) | 80 (86.4%) | 81 (88.4%) | 80 (86.4%) | 82 (89.3%) | 78 (81.6%) | 74 (79.6%) |
| CDF at 13, 16 or 18 (n=242) | 232 (95.9%) | 229 (94.6%) | 212 (87.6%) | 213 (88.0%) | 214 (88.4%) | 218 (90.1%) | 208 (86.0%) | 209 (86.4%) |
